# Review of the Report of the Chairman of the Bureau of Materia Medica of the American Institute

**Published:** 1884-05

**Authors:** P. P. Wells

**Affiliations:** Brooklyn, N. Y.


### HOMŒOPATHIC PHYSICIAN,

A MONTHLY JOURNAL OF MEDICAL SCIENCE.

"If our school ever gives up the strict inductive method of Hahnemann, we are lost, and deserve only to be mentioned as a caricature in the history of medicine."—CONSTANTINE HERING.

Vol. IV. MAY, 1884. No. 5.

### REVIEW OF THE REPORT OF THE CHAIRMAN OF THE BUREAU OF MATERIA MEDICA OF THE AMERICAN INSTITUTE, 1883.

"Leave your damnable faces and begin."—*Hamlet.*

In the March number of volume IV of THE HOMŒOPATHIC PHYSICIAN, we endeavored to forecast the work of this Bureau for 1884. We now propose to deal with its actual work done in 1883. In pursuance of this purpose we propose a few briefest comments on some of the utterances of the Chairman of this Bureau, as found in the *Transactions* of the year above named, and shall begin with one in the conclusion of the introductory remarks, accompanying synopses of recommendations from the different members of the Bureau—the subject being, "*A Model Materia Medica.*" After giving abstracts of these recommendations, the Chairman is found rather sadly and perhaps disappointedly saying:

> "Since nearly thirty years ago * * * I have been painfully sensible of the faulty methods and spurious results characterizing our drug experimentation, and have earnestly advocated the reproving of our medicines as affording the only chance for a *materia medica pura.* But as I could not, by myself, organize and maintain a college of provers, and as the profession, blind to the advantages to be gained or careless of the evils to be shunned, would not second my effort," etc.

Just so, Doctor. And don't you see the obvious and sufficient reason for this? The profession, at least the best part of it, has not believed one word of all this bosh of *advantages* and "evils to be shunned," you have been so earnest in asserting all these years, and it is quite conceivable, if there may have been some who have been in accord with your judgment of our materia medica as to its impurities, they may, at the same time, have failed to recognize in your utterances any especial fitness for the office of purifier, and so have failed to give you the support you needed. And then it is quite certain our best men have felt no want of this re-proving, which has apparently been so great a trouble to you these "thirty years." They have only been successful and happy in healing the sick with the help of this alleged "impure" materia medica; indeed, they have been so successful and happy that they have little or no confidence in your allegation.

But the subject of the report before us is "A Model for Materia Medica." A noble subject, truly, and when found mankind may well rejoice in it. Let us look at some of its utterances on this subject and see, if we can, what there is to be gathered from these of promise of our hastily realizing this grand desideratum. The first intimation of a plan for this "model" is thus given:

> "It was intended to draw out illustrations of different methods of culling from our vast displays of drug symptoms the characteristic and most important; and hence the proposition *to bring the pathogenesis of* EACH DRUG, *on an average, down to five pages.*"* * * *

Italics, etc., ours. This is certainly as remarkable a proposition as can well be imagined to have come from a body of eleven men of average intelligence. This limitation of space, as it is the first peculiarity of the proposed "model" mentioned, may be supposed to be considered by our Bureau of prime importance. The idea, taken with other utterances of this report, would seem to be, when dealing with drugs which are credited with a record too voluminous to be brought within the compass of the regulation "five pages," to vote the surplus either spurious or superfluous, and all difficulty is disposed of. We shall, by and by, examine some other utterances of this report with a view to learn from them, if we may, what are the qualifications of this Bureau, and especially of its Chairman, for this work they have had committed to them.

---

* The one idea of *excellence* of those who would give us this "model," would seem to be contained in *contraction of size* of the work. In this we are reminded strongly of the story of the Dutchman, who had much admiration for the talents of his brother, and proved his superior greatness by declaring he "had written a book as *thick as that cheese.*" The model is to be *small.*

"A careful sifting out of chaff and study of characteristics in ways illustrated in this country as well as in England, will bring us a condensed materia medica more useful to the practitioner than the cumbersome works now claiming to be complete."

Do the "ways" refer to the "sifting," or to the "study"? If to the former, then we are justified, we think, in inquiring, Why not cease this everlasting twaddle-talk of "chaff," and begin to "sift"? * These men, notably this Chairman, after all this "near thirty years'" talk of *chaff*, has not even begun to *sift*. We know no indication other than his perpetual insisting that there is much *chaff*, of any qualification in him for this work of *sifting*, and this certainly does not go far in proof.† He has not, so far as we know, given any evidence that he knows "chaff" from wheat. We remember a notable occasion, some years ago, when he, in his zeal on *chaff*, characterized one recorded fact as

---

* There were, some years since, in Vienna, a band of intelligent, earnest, honest men, who seem to have suffered from the pains and anxieties which our Chairman says have been so great a trouble to him for so many years. They did not believe in potentization, nor in the genuineness of the "vast displays of drug symptoms" they found in our materia medica, and what did they do? Did they *talk* thirty years of these pains to an unsympathizing audience? Not at all. They only went to work and re-proved some of our most important remedies, and were cured of their pains and their skepticism by this honest work;—of their pains by finding the records of previous provings so beautifully confirmed, and of their skepticism by finding wealth of symptoms from doses of potentized drugs, when the crude had given none. It was *work* which cured them, and this against their will, and not *talk* at all. They had no "college of re-provers," nor was any one of the worthy band endeavoring, perhaps "painfully," to pose before Austria and the world as a possible "president" of such a piece of folly. Perhaps this last fact may explain why they worked successfully while our Chairman has only talked. These *working* men found nothing to strike or "sift" out. They *added* much valuable matter to records already greatly extended, because they found many new *facts*, and had no "five pages" standard into the limits of which all records of each drug were to be brought.

† Whence came the license of this Chairman for this "thirty years'" abuse of our materia medica? Not from any useful labors in its interests. He has, so far as we know, added not one to the great sum of its facts. He has in no way or manner, again we say, so far as we know, helped any one to a better understanding of it, or to a more ready use of its facts in their practical duties. He has done nothing but defame this most important work of which he has all this time been so ambitious of being constituted its supreme judge. Did he forget that the common sentiment of mankind, even in less important matters, has demanded of those who were to act as judges of them that they should come to their judgment without *prejudice?* This sentiment, if the world should ever be foolish enough to institute a "*college of re-provers*," will be fatal, or should be so, to our Chairman's ambition to stand at its head. For it is certain that in all relating to our materia medica, prejudice, as compared with any knowledge he may have displayed of it, is so far in excess that the knowledge can hardly be seen at all.

of this worthless class, which an abler man at once recognized as a most important fact, not "chaff" at all, but *wheat* of the soundest kind, and intimately related as curative to the initiatory stage of apoplexy. The difference was, our Chairman ridiculed the fact and called it *chaff*. Our other friend could see its true character, and use the so-called *chaff* and cure important sicknesses with it. The trouble with our Chairman was, "he didn't know." Is this the "way" of "sifting" which is to give us our better materia medica?

But, perhaps, here is a shadow of the *way* he had in mind when he commended the *sifting* to be done.

"In the first place, as I took pains to show in my paper, for the World's Convention at Philadelphia, in 1876, a large number of drugs have been presented in our works on materia medica which have had no proper proving, or have shown little or no influence on the human organism, certainly not such as to give them rank as useful remedies."

"A large number." What are they? And how is it known they were not properly proved?* In view of his Niagara blunder of ten years ago, it can hardly be but that such *assertions* will be questioned.

"Many symptoms have been recorded by provers whose conditions or circumstances were such as to make it very doubtful if they were at all drug effects."

What does he know of the "conditions or circumstances" of the provers whose work he here calls in question? We believe we hazard nothing in saying he can know but very little. And yet this little is to give us our improved materia medica.

"Many symptoms have been noted as drug effects where doses were taken in which there would not possibly be any drug influence."†

How does this man know this? We have an impression that Omniscience only knows the limit of the possible or impossible in this matter of drug action. Our Chairman may as well know now that the world will not concede to him this attribute. They are very likely to tell him,

---

* Is this because they have not passed under the revision of a "college of re-provers," which as yet has no existence, and received the approval of this shadowy authority?

† No "drug influence" was just what the Vienna re-provers thought *till they tried it*, and so found their mistake. We commend their example to our Chairman as eminently worthy of following by him and all others who have come to *a priori* conclusions of "*no drug influence*."

those who are most intelligent in this matter of drug action, that he knows just nothing at all about the impossibility of it. And in this he is only like the rest of us.

"Many symptoms have been attributed to medicines that were really due to the lively imagination and undue ambition of self-appointed provers."*

How many and what are these symptoms? How many of these provers has our Chairman known personally that he should dare to characterize them and their work thus dishonorably? If the plain truth were known, we venture the guess that it would appear he knows little of either, and we should be left to the conclusion that this charge has had no other origin than our Chairman's imagination, whether "lively" or otherwise.

"A large number of symptoms stand in the materia medica which have occurred in but one prover, and in that prover but once."

How many? And what then? If we are not mistaken, such symptoms have led to effective curing agents more than once, showing they were facts all the same, by that most conclusive of all evidence, their clinical verification. It is not whether a symptom has appeared in only one or many provings; the only question of importance is: Is the symptom a part? Will our Chairman tells us how he knows these he has discredited are *not facts?* Repetitions, either in many persons, or in one, do not create facts. They may confirm our confidence in them. And would our Chairman exclude facts from our materia medica on no better ground than his want of personal confidence in them? or from a fear of a "pathogenisis of some drugs" extending beyond the prescribed limits of five pages? If so, who gave him this authority? Is this another of the "ways" of "*sifting*" by which we are to come to our "*model*" materia medica? If so, then let us be thankful our Chairman is not yet at the head of any "college of re-provers."

"Many symptoms in the sick appearing for the first time after the administration of a remedy have been recorded as drug effects."

Does the Chairman know these symptoms were not the effects of the drug? The so-called clinical symptoms scattered through our materia medica may some of them have had this origin for aught we know, but, we are quite confident, not all of them.

---

* *I.e.*, provers who belong to no "college" and have been so poor as to have no president for their guide with that omniscience which at once and infallibly detects and discriminates drug effects from the workings of their seductive "ambitions" and "imaginations."

They constitute a very important part of our drug record, and were placed there by our best men, and only after such consideration as satisfied them of the truth and value of these symptoms in therapeutics. We know of no superior intelligence in our Chairman which warrants his revision of their work. These symptoms are often sadly missed from our latest great work of drug records, from which they have been excluded. They are a loss, but only a small one, in comparison, we judge, with that which will be regretted as the outcome of the more liberal exclusions shadowed in the plan of our proposed "*model.*"

"Symptons noted in cases of poisoning have been attributed to the poisonous drug taken, without due consideration of the influence exerted by antidotes and other causes at the same time operative."

How "without due consideration"? What does our Chairman know of the "consideration" these symptoms have received which is here denied an existence? These symptoms have been placed in the record by men of knowledge of drug action more than others, and to suggest an equality of our Chairman with them in this respect would be a flattery far transcending modesty, decency, or truth. The men who have wrought out for us our materia medica were not given to passing their work into print without a consideration of no common carefulness. Our Chairman has, as compared with them, no superior knowledge of sources of error in making up the record, nor a more careful conscience for their avoidance. We speak now of the earlier laborers in this great work. It is quite possible some of the more modern may deserve all our Chairman's suspicion and criticism. If there be such, we have nothing to say in their defense. And, further:

"When all worthless and unproved drugs, and all symptons, which are not drug effects, are omitted there will be a wonderful shrinkage in our materia medica."

No doubt. But who is to decide as to the worthless drugs, and by what standard are they to be adjudicated as such? As to unproved drugs, they can present no difficulty. They may all be dismissed to the limbo of the impracticable. But the worthless—who shall declare them? Not every one who decries our materia medica is equal to the task, as we have often shown, and we are nearly certain that any man who is equal to it will feel a great shrinking from the undertaking. What shall be the standard by which he is to be governed in his decisions? We know of none. If we adopt the rare call for the clinical use of a drug or that it has not been called for at all in our experience of more than forty years of homœopathic practice, it would seem, perhaps, that such a drug might be excluded without loss. But it is not so, as was recently proved when such a drug brought to us the rare experience of a violent case of scarlet fever, cut short *in limine*, by a single dose of it. There was no other member of the materia medica which met the demands of this case, and this one did and cured. Suppose it had previously been excluded, who can say what would have been the course and termination of the case? Evidently rarity of use is no ground for exclusion. Then what is? Let the ambitious man answer this before he proceeds to any wholesale "sifting" out. But further:

"Genuine drug symptoms are not all of equal value, and when by much scrutiny and comparison we are able to distinguish those of the *gratest value* to arrive at the *characteristic* and *essential*, then may we hope for a condensed materia medica worthy of the name, etc."

Certainly, there is a sense in which it may be said "Genuine drug symptoms are not of equal value." If by this it be meant that those symptoms of the drug most frequently met in counterpart in sicknesses to be healed are of the first consideration, we have nothing to say against it. But we do say that whoever thinks this greater value is to be discovered by any amount of "scrutiny and comparison" is only deceiving himself. This kind of greater value is only learned by clinical experience of the fact. But there is another reason for assigning greater value to some symptoms than to others, and this is a fundamental one. That in the drug is of greatest value, therapeutically, which is *most like* the symptomatic elements of the case in hand to be cured. This can only be found at the side of the sick-bed, and not by any amount of "scrutiny and comparison" elsewhere, in endeavors to "condense" our materia medica by exclusion. Then there is another sense in which "genuine drug symptoms" *are* of *equal* value. All such are facts, and, as before the prover, are to be alike recorded with care, for it may well turn out that the fact, when recorded, judged as of least worth, may be found to be just that which is *most like* in the first case of sickness calling for its use, and then for that case it becomes most valuable of all. But this discovery is not learned by "scrutiny" in the interest of endeavors to *condense* our record. This, if based on *a priori* judgment, will inevitably be found excluding most valuable matter, so inflicting loss rather than conferring benefit. And further still and more remarkable, we read:

"It is well known to every thoughtful student of materia medica that each drug must primarily impress a certain tissue and disturb the functions of a certain organ, and that when not prevented by some other morbific* or pathogenic agency, the character of its impression must be the same in all cases. The inception and progress of the drug disease undisturbed must be essentially the same in all persons; and the symptoms indicating its point of attack, its line of march, and its results cannot differ widely in the members of the human family. *Upon this uniformity of drug effects depends the whole fabric of Homœopathy.*"

Any student of materia medica, whether very thoughtful or not, must know that this paragraph is very far from the truth, unless its saving clause of "morbific or pathogenic agency" be carried farther in its meaning than a just sense of the words will warrant. There has not been in the experience of provers, and is not now in the records of their experiences, any such "uniformity" as is here set forth, and Homœopathy is in no sense or degree dependent on such a basis, which has no foundation save in the imagination of our Chairman. Homœopathy depends and rests on facts wholly, and not in the least on any man's fancy. So far is this alleged "uniformity" from fact, that the very reverse is true, and hence the necessity of provings of the same drug by persons of all sexes, ages, temperaments, conditions, and idiosyncrasies, in order to secure to the record the whole scope of the possible effects of the drug. These have all been recognized by our masters in the art of drug proving as modifiers of drug action in individual provers, and hence have been destructive of the alleged "uniformity" in drug action. It is certain no one of these can be justly forced into either category of "morbific or pathogenic action" for the salvation of our Chairman's declaration from the domain of sheer nonsense. It is singular, but perhaps not unexpected, how so nearly every utterance of this Chairman has demonstrated his utter unfitness as a reformer of materia medica, as well as to preside over a so-called "college of re-provers." The last quoted paragraph adds one more instance of evidence to those already before us, proving this incompetency, which, we mav surely be pardoned if we suggest, it was not needed. And again:

"If the symptoms recorded in our materia medica do not apply to the generality of mankind [of course they do], if they are to represent personal peculiarities [equally, of course they are not], and not uniform drug effects, the practical application of *similia* becomes a task more difficult and less satisfactory than out-and-out empiricism."

* The prover is supposed to be in health.

Of course, practice under and according to law is "more difficult" than any empiricism.* This has always been and will be a most difficult duty. There is none more so. But that it is less "satisfactory" we deny in sharpest terms. This statement could never have come from an honest man who had faithfully tried it. And yet this man would be a reformer of our materia medica! No wonder the first idea of reform of such a man was to *strike out!* This is easy. Any man can do it—the least qualified for the work as effectually as the best.

And then the dodge of representing the facts of our record as of "personalities" and not as "drug effects," when they have been gathered, as they rightfully were, from the experiences of these effects on individualities of all sexes, ages, temperaments, conditions, and idiosyncrasies, is hardly above contempt. This variety of proving was necessary in order to gaining a complete knowledge of the scope of the drug action, and not for a knowledge of any "personality" whatever. The variety in these "personalities" of the provers, as indicated above, represents to us variety in each, of susceptibility to the action of each drug, the record being of that action, different, perhaps, on each different susceptibility, and not the record of the susceptibility. It is very likely it required a professed reformer to conceive of so absurd an idea. The drug impresses one susceptibility in the manner in which it is its nature to do this, because of its relationship to this form of life. It affects a different susceptibility in a different manner because of the same natural relationship, and so on to the end of the series. But all are effects of the same drug, though the recorded facts of these provings are so utterly destructive of our Chairman's alleged "uniformity of drug action," and so effectually scatter his other allegation of the "dependency" of Homoeopathy on this "uniformity."

P. P. WELLS.

* This is a singular comparison. Everybody knows empiricism has *no* difficulties. That it is "*just as easy as lying*," *i. e.*, any other *kind* of lying, while practical Homœopathy is confessedly one of the most difficult of human undertakings.

